# An Enhanced Carbon Capture and Storage Process (e-CCS) Applied to Shallow Reservoirs Using Nanofluids Based on Nitrogen-Rich Carbon Nanospheres

**DOI:** 10.3390/ma12132088

**Published:** 2019-06-28

**Authors:** Elizabeth Rodriguez Acevedo, Farid B. Cortés, Camilo A. Franco, Francisco Carrasco-Marín, Agustín F. Pérez-Cadenas, Vanessa Fierro, Alain Celzard, Sébastien Schaefer, Agustin Cardona Molina

**Affiliations:** 1 Grupo de Investigación en Fenómenos de Superficie–Michael Polanyi, Facultad de Minas, Universidad Nacional de Colombia-Sede Medellín, Medellín 050034, Colombia; 2 Research Group in Carbon Materials, Faculty of Sciences, University of Granada, Granada 18071, Spain; 3 Bio-Sourced Materials Research Group, Institut Jean Lamour, UMR CNRS–Université de Lorraine, Epinal 88051, France; 4 Grupo de Investigación en Yacimientos de Hidrocarburos, Facultad de Minas, Universidad Nacional de Colombia-Sede Medellin, Medellín 050034, Colombia

**Keywords:** adsorption, carbon capture and storage process (CCS), carbon dioxide, nanofluids, nanoparticles and shallow reservoirs

## Abstract

The implementation of carbon capture and storage process (CCS) has been unsuccessful to date, mainly due to the technical issues and high costs associated with two main stages: (1) CO_2_ separation from flue gas and (2) CO_2_ injection in deep geological deposits, more than 300 m, where CO_2_ is in supercritical conditions. This study proposes, for the first time, an enhanced CCS process (e-CCS), in which the stage of CO_2_ separation is removed and the flue gas is injected directly in shallow reservoirs located at less than 300 m, where the adsorptive phenomena control CO_2_ storage. Nitrogen-rich carbon nanospheres were used as modifying agents of the reservoir porous texture to improve both the CO_2_ adsorption capacity and selectivity. For this purpose, sandstone was impregnated with a nanofluid and CO_2_ adsorption was evaluated at different pressures (atmospheric pressure and from 3 × 10^−3^ MPa to 3.0 MPa) and temperatures (0, 25, and 50 °C). As a main result, a mass fraction of only 20% of nanomaterials increased both the surface area and the molecular interactions, so that the increase of adsorption capacity at shallow reservoir conditions (50 °C and 3.0 MPa) was more than 677 times (from 0.00125 to 0.9 mmol g^−1^).

## 1. Introduction

In recent decades, climate changes have generated negative consequences such as loss of sea ice, accelerated rise of sea level, extinction of some species, drought and population displacements, among others [1,2,3,4,5]. These changes are caused by human activities, mainly through the release of greenhouse gases [4,5,6,7]. The anthropogenic emissions of carbon dioxide, CO_2_, from fossil fuel combustion and industrial processes contributed to about 78% of the total greenhouse gases emissions [8,9,10], and CO_2_ emissions have increased by 46% since pre-industrial times [11,12]. The huge CO_2_ daily emissions and their growth increase its responsibility on global climate change [12,13,14,15,16]. Raw material industries such as chemical, petrochemical, iron, steel, cement, and others, contribute with the most to CO_2_ emissions worldwide [17,18]. The advantage of CO_2_ industrial emissions is that they are fixed sources so that they can be controlled in-situ. A typical flue gas from coal-fired boilers may contain 12–14 vol% CO_2_, 8–10 vol% H_2_O, 3–5 vol% O_2_, and 72–77% N_2_ [19,20,21]. The method most frequently used at industrial levels for CO_2_ capture is absorption. However, this method has significant limitations related to solvent regeneration, composition of flue gas, CO_2_ concentration, corrosion, high-energy consumption, etc. [15,16,18,22]. Other methods are cryogenic distillation, membranes, and adsorption by porous solids. Hence, the current methods are not enough, and CO_2_ emissions continue to increase [12,17,23]. For this reason, it is necessary to broaden the portfolio of methods to reduce CO_2_ emissions significantly in the years to come.

**Figure 1:**
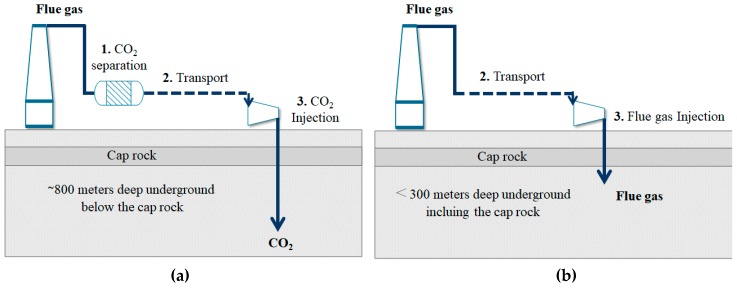
Configurations of the carbon capture and storage (CCS) process: (**a**) conventional CCS process; (**b**) proposed enhanced CSS (e-CCS) process.

The Intergovernmental Panel on Climate Change (IPCC) promotes the carbon capture and storage process (CCS), which allows the geological storage of CO_2_ emitted by the industry over the long term [10,18,24]. The CCS process might decrease CO_2_ emissions by approximately 22% in 2035 [25,26]. The CCS process has three main stages: 1) CO_2_ capture and separation from flue gas, 2) CO_2_ transport to the storage site, and 3) CO_2_ injection into deep geological storage sites, 300 to 4000 m, and 800 m on average [22]. In this case, CO_2_ capture and storage are mainly due to the inter-particle volume filling. Figure 1a presents a scheme of the CCS process.

It is estimated that 50% of the CCS projects use sandstone deposits for storage because they have technical advantages and high availability [24]. Oil and gas deposits are indeed mostly composed of sandstone, which provides a natural storage structure, with porous texture and upper and lateral seals that allow long-term storage. Carbon capture and storage have been coupled with other processes such as enhanced oil recovery (EOR) or enhanced coal bed methane production (ECBM) [24].

However, its in situ industrial implementation has been unsuccessful mainly due to the technical and economic costs associated with the separation and storage stages. In general, the cost of CO_2_ capture is 70–80% of the total CCS costs [26,27,28]. For this reason, the current research focuses mainly on increasing the CO_2_/flue gas separation capacity in order to decrease energy consumption, or on the use of waste energy, among others. This study proposes an alternative to minimize the technical and economic cost for the viability of the CCS process. Figure 1b presents the configuration of an enhanced CCS process (e-CCS), in which the stage of CO_2_ capture/separation is removed, and the flue gas is injected directly into shallow deposits, at depths less than 300 m. In this case, CO_2_ remains in a gaseous state, and the adsorption process controls the capture and storage. The density of CO_2_ is very different in gaseous or supercritical conditions, which affects the amount of CO_2_ stored in the e-CCS process. For this, it is necessary to add a modifying agent to the surface of the porous medium in order to improve the adsorption capacity and the CO_2_ selectivity, since the adsorption (capture/storage) process is done underground. In addition, the modifying agent should not affect the naturally porous structure of the deposit to avoid operational problems.

Nanostructured materials constitute a vast and active field of research in various areas, due to their intrinsic characteristics that can be adjusted depending on the foreseen application, such as porosity, structure, molecular affinity, high surface-area-to-volume ratio, high surface activity, dispersion capacity, and optical and electronic properties, etc. [29]. Nanoparticles have been used to increase the recovery of oil and gas by modifying the physicochemical properties of the reservoirs [30,31], and to modify reservoirs’ wettability, asphaltenes adsorption, catalysis and stabilization of fines in reservoirs, among others [30,32,33,34,35]. The obtained results demonstrate their effectiveness in achieving this objective without obstruction of the porous media. In this way, nanospheres can be an option for the modification of the surface in shallow reservoirs for the e-CCS process. Although nanoparticles have been evaluated for the conventional CCS process in the form of a nanofluid used to improve CO_2_ transport in saline aquifers [36], they have not been used for rock modification and improvement of the CO_2_ storage capacity, such as in the case of the proposed e-CCS process, to the best of our knowledge.

Many adsorbents for CO_2_ capture have been reported, such as carbon materials, metal organic frameworks, zeolites, alkali metal carbonates, etc. [16,37,38,39], but not widely at the nanoscale level, with spherical structures allowing their application in geological deposits. Many carbon nanostructures have been evaluated for CO_2_ capture, among them nanofibers, nanosheets, and nanotubes, leading to adsorption capacities ranging from 0.26 to 4.15 mmol g^−1^ under atmospheric conditions [16,40,41,42,43,44,45]. However, these materials are not applicable to reservoirs due to their structure and dimensions that might affect the nature of the reservoir’s porous structure. Carbon nanospheres, thus, appear to be the best choice for the e-CCS process. Wang et al. [16] analyzed different adsorbents and concluded that carbon materials are one of the best options, mainly due to their low cost, high surface area, adjustable porous texture, and easy surface functionalization [16,37,38,39]. Chen et al. [46] reported hollow carbon nanospheres with a CO_2_ adsorption capacity of 3.65 mmol g^−1^ under atmospheric conditions [46], which is similar to what was reported for other nanomaterials such as carbon fibers, carbon nanosheets, carbon nanotubes, metal organic frameworks, zeolites, alkali metal carbonates, etc [16,37,38,39]. For CO_2_ adsorption applications, a high surface area is essential as well as basic functionalities, due to the acidic character of the CO_2_ molecule. Therefore, high nitrogen content in the adsorbent allows obtaining a basic nature and enhances the adsorption capacity and selectivity. Some authors suggested increasing the nitrogen content by impregnating the materials with amines like the materials commonly called supported amine material, which consist of a porous support onto which an amine is attached or immobilized [16,37,38,39,47]. However, treatment with amines can obstruct the microporosity of the nanomaterial, thereby decreasing its adsorption capacity and increasing its final cost. Also, in some cases, like samples containing grafted primary and tertiary monoamines, the material could be deactivated in the presence of oxygen-containing gases [47]. Thus, it is desirable to incorporate nitrogen groups during the material’s synthesis. Hence, amine or amino acid functional groups are grafted on the surface of the support during the synthesis process instead of physically dispersed in the pores after synthesizing the support material [48]. 

The main objective of this manuscript was, therefore, to experimentally study the possibility of improving the CCS process through nanotechnology. For this, carbon nanospheres with different structures were synthesized, characterized, and used to impregnate sandstone using different mass ratios of nanoparticles to sandstone. The CO_2_ adsorption process, as well as its thermodynamic parameters, were evaluated under atmospheric and high-pressure conditions.

## 2. Materials and Methods

Two different carbon nanostructures were synthesized using either a sol-gel method or a solvothermal method. The synthesized nanostructures were labeled and synthesized as follows:

(1) CN.LYS: Carbon nanospheres obtained from a sol-gel method, using resorcinol/formaldehyde as carbon precursor and L-lysine as catalyst and nitrogen precursor. 

(2) CN.MEL: Carbon nanostructures obtained from a solvothermal method, using carbon tetrachloride as carbon precursor and melamine as nitrogen precursor. 

Both CN.LYS and CN.MEL were characterized in order to select the best material, considering the nanometer size, adsorption capacity, lower technical and economic cost, and method of synthesis. Ottawa sandstone was used as porous medium and was impregnated with the best nanomaterial at different percentages. The performances of the materials were evaluated by CO_2_ adsorption at atmospheric pressure and at 0 °C, and by varying pressure and temperature conditions. The detailed procedures are presented below.

### 2.1. Materials and Reagents

For the synthesis processes, the following reagents were used, all from Sigma–Adrich, St. Louis, USA: carbon tetrachloride (≥99.9%), melamine (99%), formaldehyde (37%), resorcinol (≥ 99%), L-lysine (>98%), sodium dodecylbenzene sulfonate (SDBS), and deionized water.

For cleaning, drying, and carbonization, the following chemicals were used, all from Sigma–Adrich again except N_2_: acetone (99.9%), ethanol (99.5%), hydrochloric acid (37%,), *tert*-butanol (≥99.5%), and N_2_ (high purity, grade 5.0). Clean Ottawa sandstone and sandstone from a real reservoir were used as porous media. The real sandstone was obtained from a Colombian oil field, which allows evaluating the real rock that might be used to implement the e-CCS process in depleted oil fields.

### 2.2. Synthesis of Nanomaterials

#### 2.2.1. CN.LYS Synthesis

The process was adapted from Yong–Rong et al. [48], changing the lysine concentration, the reaction time and the resorcinol/water molar ratio. A solution (S1) of resorcinol/formaldehyde in a 1:2 molar ratio, and deionized water was stirred at 25 °C for 1 h. In parallel, a solution (S2) of L-lysine and deionized water was stirred at 60 °C for 1 h. The molar ratio of resorcinol/L-lysine was 1:0.16. Subsequently, the solutions S1 and S2 were mixed at 60 °C for 1 h to obtain the solution S3. The latter was maintained at 25 °C for 20 h to benefit from the natural precipitation of the nanomaterial. Finally, the obtained polymer was dried at 120 °C for 12 h and carbonized under N_2_ flowing at 60 mL min^−1^, using a tubular furnace. The temperature was increased up to 800 °C at a rate of 1 °C min^−1^, and the final temperature was held for 5 h. The employed molar ratios of resorcinol/water (for S1) were 1:2778 (without dilution), 1:5556 (dilution 1), and 1:11112 (dilution 2) for obtaining the CN.LYS1, CN.LYS2, and CN.LYS3 materials, respectively. Different molar ratios of resorcinol to water were used to reduce particle size.

#### 2.2.2. CN.MEL Synthesis

The CN.MEL synthesis was adapted from Bai et al. [49] by dissolving 2 g of melamine in 120 mL of carbon tetrachloride [49]. This solution was put in a stainless-steel autoclave (Parr Instrument, Illinois, USA) with a capacity of 200 mL and introduced into an oven (Thermo Fisher Scientific, Massachusetts, USA) at 250 °C for 24 h. The synthesis was carried out under auto-generated pressure. Subsequently, the carbonaceous material that formed was separated from the solution, and was cleaned with acetone, ethanol, and finally 0.1 mol L^−1^ HCl. A mixture of nanospheres and fibers (formed by aggregation of nanospheres) was obtained (Gel.MEL1).

To obtain N-rich carbon spheres, the Gel.MEL1 was coated with a mixture of resorcinol and formaldehyde in a 1:2 molar ratio. Initially, the gel was stirred with sodium dodecylbenzene sulfonate (SDBS) at 25 °C for 18 h to promote the subsequent interaction with resorcinol/formaldehyde. After 18 h, the gel/SDBS was mixed with resorcinol/formaldehyde (in proportions of 0.37 g of nanoparticles per 1 g of resorcinol) and put in a stainless-steel autoclave at 130 °C for 24 h. In order to maintain its porous texture, the gel was dried by freeze-drying. First, the material was impregnated with *tert*-butanol for three days. Then, the impregnated material was frozen at −5 °C and lyophilized for two days until the *tert*-butanol was entirely removed (Gel.MEL2). Finally, the lyophilized gel was carbonized under N_2_ flow (60 mL min^−1^) in a tubular furnace (Thermo Fisher Scientific, Massachusetts, USA). The temperature was increased at a heating rate of 1 °C min^−1^ up to 700 °C, and the latter temperature was held for 6 h.

#### 2.2.3. Impregnation of Sandstones 

The sandstone was impregnated to decorate the rock surface with the nanoparticles and improve the surface area and molecular interactions. Ottawa sandstone (SS) was impregnated with CN.LYS2 at mass fractions of 0.01, 0.1, 1, 5, 10, and 20% by immersion and soaking [50]. Initially, a nanofluid composed of nanoparticles and deionized water was sonicated at 40 °C for 4 h. Subsequently, the SS was introduced in the nanofluid at 60 °C for either 6 h at 600 rpm or for 24 h without stirring.

The latter method better mimics the reservoir conditions in which the porous medium might be impregnated. Finally, the impregnated material was dried at 110 °C for 12 h. The same procedure was followed for impregnating the sandstone from a real reservoir (RS) but using only a mass fraction of 10 and 20% of CN.LYS2 to RS and 24 h of soaking.

### 2.3. Characterization of the Nanomaterials

The following procedures allowed characterizing the physicochemical characteristics of the materials, essential for a good understanding of the results. For e-CCS application, nanoparticles must have a nanometer size, a spherical shape, a high surface area, and a high nitrogen content.

#### 2.3.1. Size and Structure of Nanomaterials

Different techniques were used to evaluate the particle size distribution: scanning electron microscopy (SEM) was used to obtain the dry particle size, size distribution, and morphology of CN.MEL, whereas transmission electron microscopy (TEM) was used to analyze the dry particle size, size distribution, and surface characteristics of CN.LYS. The CN.LYS showed a different porous structure after dilutions, which was observed by N_2_ adsorption (at −196 °C). Further, TEM was used to characterize the structure after dilutions to analyze the causes of pore structure modification. Scanning electron microscopy analysis was also carried out to analyze the distribution of carbon nanoparticles on sandstone after impregnation. The observations were carried out by means of a JSM-7100 emission electron microscope (JEOL, Nieuw-Vennep, The Netherlands), a GEMINI-LEO1530 VP FE-SEM emission electron microscope (Carl Zeiss, Cambridge, UK), and a Tecnai F20 Super Twin TMP transmission electron microscope (FEI, Hillsboro, USA).

Dynamic light scattering (DLS) was carried out by means of a NanoPlus-3 zeta/nanoparticle analyzer (Micromeritics, Norcross, USA) at 25 °C in a glass cell (capacity of 0.9 mL), which was used to obtain the mean particle size of nanoparticles dispersed in a fluid, which hydrate and interact with each other. The mean particle size was calculated from the diffusional properties of the particle indicating the size of the hydrated and solvated particle. For this purpose, a nanoparticle solution, 10 mg L^−1^, was dispersed in water or ethanol and sonicated for 6 h before analysis. Particles suspended in a liquid have a Brownian motion due to the random collisions with solvent molecules. This motion causes the particles to diffuse through the medium. The diffusion coefficient, *D*, is inversely proportional to the particle size or hydrodynamic diameter, *d*, according to the Stokes–Einstein equation:

(1) $$D\;=\;\frac{k_{B}\;T}{3\;\pi \;\eta \;d}$$

where, *k*_B_ is Boltzmann’s constant, *T* is the absolute temperature, and *η* is the viscosity.

#### 2.3.2. Porous Structure of Nanomaterials and Sandstone

All materials were characterized by N_2_ and CO_2_ adsorption at −196 °C and 0 °C, respectively, using a 3-Flex manometric adsorption equipment (Micromeritics, Norcross, USA). The total adsorbed volume (*V*_0.95_) was taken as the physiosorbed volume of N_2_ at a relative pressure *P*/*P*_0_ = 0.95. The Brunauer–Emmett–Teller (BET) model was applied to obtain the BET area (*A*_BET_). Micropore volume (*V*_mic_), average pore size (*L*_0_), and CO_2_ adsorption energy (*E*_ads_–_CO2_) were obtained by application of the Dubinin–Radushkevich equation. The mesopore volume (*V*_meso_) was obtained through the Barrett–Joyner–Halenda (BJH) model.

#### 2.3.3. Chemical Composition of Nanomaterials and Sandstone

The chemical characterization was carried out by carbon, hydrogen, oxygen and nitrogen (CHON) analysis for nanomaterials, and by Fourier transform infrared spectroscopy (FTIR) for sandstone. An IRAffinity-1S FTIR spectrometer (Shimadzu, Columbia, USA)was operated at room temperature using potassium bromide in a KBr-to-material ratio of 30:1 (% w/w). The impregnation percentages of nanoparticles on sandstone were corroborated by thermogravimetric analysis (TGA) (TA Instruments, New Castle, USA). For this, the sample was burned under an air atmosphere at 10 °C min^−1^ up to 800 °C.

#### 2.3.4. Rheological Analysis of CN.LYS Synthesis Solutions

The stability of the synthesis solutions was evaluated at different concentrations of CN.LYS (CN-LYS1, CN.LYS2, and CN.LYS3). For this purpose, a Kinexus Ultra+ rheometer (Malvern Panalytical, Malvern, UK) utilizing a concentric cylinder sensor equipped with a Peltier cell for temperature control was used. The tests were first carried out by varying the shear rate from 1 to 250 s^−1^ in order to define the adequate shear rate (50 s^−1^). The test conditions were carried out to mimic the real synthesis conditions in terms of temperature and stirring. The process started at 60 °C for 1 h, after which the temperature was controlled to simulate the natural cooling process at 57, 45, 37, and 34 °C until the viscosity reached a constant value. Only 30 mL were needed for the test, while the current reaction was carried out in 1.8 L.

#### 2.3.5. Dispersion of Nanoparticles in Solution

The electrophoretic light scattering (ELS) technique was used to evaluate the surface charge of the particles and their dispersion stability at 25 °C in a NanoPlus-3 zeta/nanoparticle analyzer (Micromeritics, Norcross, USA). In this test, several nanoparticle suspensions were prepared at 10 mg L^−1^, with a pH adjusted between 2 to 12 by adding solutions of 0.1 mol L^−1^ HCl or 0.01 mol L^−1^ NaOH, and then subjected to analysis. The zeta potential was calculated using the Smoluchowski equation, derived from the calculation of the Doppler effect.

(2) ζ = η U / ε

(3) $$U\;=\;\frac{V}{E}$$

(4) $$\Delta \nu \;=\;2V\;n\;\sin\left(\frac{\theta }{2}\right)/\lambda$$

where *ζ* is the zeta potential, *η* is the viscosity of the fluid (water), *U* is the electrophoretic mobility, *ε* is the permittivity, *V* represents the speed of movement of the particles, *E* is the electric field, ∆*ν* is the Doppler effect, *n* is the index of refraction, *θ* is the angle of detection, and *λ* is the wavelength of the incident light.

### 2.4. Adsorption Tests at High Pressure

The adsorption tests carried out below atmospheric pressure were described in sub-Section 2.3.2. At high pressure, the CO_2_ adsorption capacity was evaluated in two different conditions: (i) under pure CO_2_ in a manometric device (up to 3.0 MPa) and (ii) under a CO_2_/N_2_ flow in a gravimetric device (up to 2.6 MPa). The details of each protocol are presented below.

#### 2.4.1. Adsorption at High Pressure for Pure CO_2_–Manometric Device

The carbon nanospheres (CN.LYS2), sandstone, and impregnated sandstone (at mass fractions of 10 and 20%) were investigated in High Pressure Volume Analyzer, HPVAII-200 (Micromeritics, Norcross, USA) at 0 °C, 25 °C, and 50 °C and at pressures from 3 × 10^−3^ up to 3.0 MPa. In order to have enough total surface area for adsorption and to minimize measurement errors, the amount of each material inside the sample holder was around 0.5 g for nanoparticles, 1.5 g for impregnated sandstone, and 14 g for sandstone. The contribution of the empty sample holder was systematically measured and subtracted to all data to improve accuracy. The isosteric heat of adsorption, *Q_ST_*, was calculated using the isosteric method with the Microactive software (from Micromeritics, Norcross, USA) from three adsorption isotherms at 0, 25, and 50 °C, based on the Clausius–Clapeyron equation [51]:

(5) $$-\frac{Q_{ST}}{R}\;=\;\frac{\partial \;ln\;(P)}{\partial \;(1/T)}$$

where *R* is the universal gas constant (8.314 J mol^−1^ K^−1^), *P* is the absolute pressure, and *T* is the temperature.

The excess adsorbed CO_2_ amount (*N*_exc_, g_CO2_.g_adsorbent_^−1^) was equal to the absolute adsorbed CO_2_ amount (*N*_ads,_ g_CO2_.g_adsorbent_^−1^) minus the product of gas density in the bulk phase by the volume of the adsorbed phase. The values provided by the HPVA device were obtained on an excess basis, and therefore, the absolute amounts had to be determined as follows [52]:

(6) $$N_{ads}{\;=\;N}_{exc\;}\left(1+\frac{{P+M}_{CO2}}{{Z\;\rho }_{liq}\;R\;T}\right)$$

where *M*_CO2_ is the molecular weight of CO_2_ (44.013 g mol^−1^), *Z* is the compressibility factor at the considered pressure and temperature, and *ρ*_liq_ is the density of liquid CO_2_ (1032 × 10^3^ g m^−3^).

The isotherms were fitted with the Sips and Toth models, which take into account multilayer adsorption. Table 1 presents the equations for each model [53,54,55]. *K*_S_ and *K*_T_ represent adsorption equilibrium constants for the Sips and Toth models, respectively, and the *n* and *t* parameters indicate the heterogeneity of the system for the Sips and Toth models, respectively. The heterogeneity may originate from the solid structure, from the solid energy properties, or from the adsorbate [54]. The *n* or *t* parameters are usually greater than unity, and when they are the unit, the models assume the Langmuir equation [54].

(7) $$N_{ads}{\;=\;N}_{m}\frac{\left(K_{S}\;P\right){\;}^{1/n}}{1\;+\;{\left(K_{S}\;P\right)}^{\;1/n}}$$

(8) $$N_{ads}{\;=\;N}_{m}\frac{\left(K_{T}\;P\right){\;}^{1/t}}{1\;+\;{\left(K_{T}\;P\right)}^{\;1/t}}$$

#### 2.4.2. Adsorption at High Pressure for CO_2_ and N_2_–Gravimetric Device

The CN.LYS2, sandstone, and impregnated sandstone CO_2_ isotherms (with a mass fraction of 20% of nanoparticles) were investigated using a HP TGA 750 thermogravimetric analyzer (TA Instruments, New Castle, USA) at 50 °C and high pressure from 0.03 to 3.0 MPa for CO_2_ and N_2_. This device was equipped with a magnetic levitation top-loading balance, which made it possible to achieve high accuracy and reduce the volume of the system. The amount of each material put inside the sample holder was around 15 mg for nanoparticles, 40 mg for sandstone, and 40 mg for impregnated sandstone, to have enough total surface area for adsorption. The contribution of the buoyancy effect was manually subtracted from the data using blank tests carried out in the same conditions but with an empty sample holder. From the adsorption results of each N_2_ and CO_2_ isotherm, it was possible to predict the selectivity by applying the ideal adsorbed solution theory (IAST), which allows estimating the competitive adsorption of the compounds in a mixture of gases as the flue gas. Based on the literature, a model flue gas comprising 80% N_2_ and 20% CO_2_ was selected. The IAST was implemented in a Python routine (package-pyIAST from Simon et al. [56]).

## 3. Results and Discussion

The results are divided into two main sections: (a) materials characteristics (nanoparticles and sandstone) and (b) study of the interaction between CO_2_/nanoparticles/sandstone by adsorption isotherms under different operation conditions (T, P).

### 3.1. Materials Characteristics

**Figure 2:**
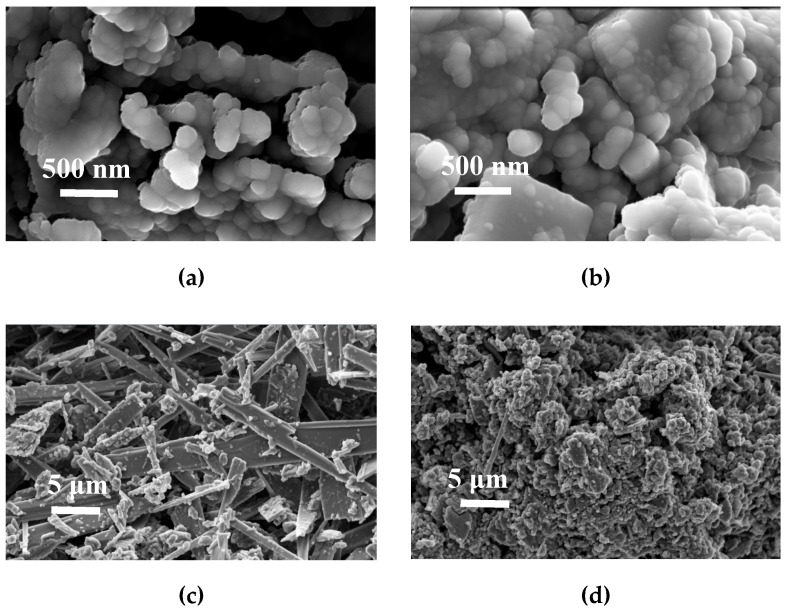
SEM images at 5 kV of carbon nanospheres from melamine (CN.MEL) before resorcinol/formaldehyde coating and final pyrolysis: (**a**,**b**) nanospheres and build fibers and blocks; (**c**) area with structures in the form of fibers and blocks, and (**d**) distribution of nanospheres, fibers, and blocks.

The morphology of the carbon materials obtained from melamine and carbon tetrachloride was very heterogeneous. Figure 2a,b presents SEM images of two different zones wherein more or less agglomerated nanospheres can be observed (Figure 2b). When the images are observed at lower magnification, it can be noticed that some areas have fiber and block morphologies (Figure 2c), while other areas have nanospheres/microspheres morphologies (Figure 2d).

After coating with resorcinol/formaldehyde, the hydrodynamic diameter of the CN.MEL particles was higher than the limit of detection of the equipment (10 μm). For the e-CCS process, this material might, thus, induce technical problems, due to the possible obstruction of the naturally porous structure of the rock. The ultimate analysis (Table 2) shows that this material had a nitrogen content close to 50% before coating and carbonization (Gel.MEL1), but of only 9.2% after coating with resorcinol/formaldehyde (Gel.MEL2) and 2.2% after carbonization (CN.MEL). Therefore, CN.LYS and CN.MEL materials exhibited similar nitrogen contents. Some N-rich carbon materials, reported in the literature, have nitrogen content close to those obtained in this work [16]. The oxygen content was measured independently from carbon, hydrogen and nitrogen content. 

The Gel.MEL1 (49.1% of nitrogen) was submitted to CO_2_ and N_2_ adsorption (at 0 °C and −196 °C, respectively) but it was impossible to obtain the corresponding isotherms, possibly because of its too low surface area. It is well known that the pyrolysis step induces a significant increase of narrow porosity by elimination of volatile species. However, when the Gel.MEL1 is directly pyrolyzed, most of the material undergoes decompositions and the yield is very low (< 5%).

**Figure 3:**
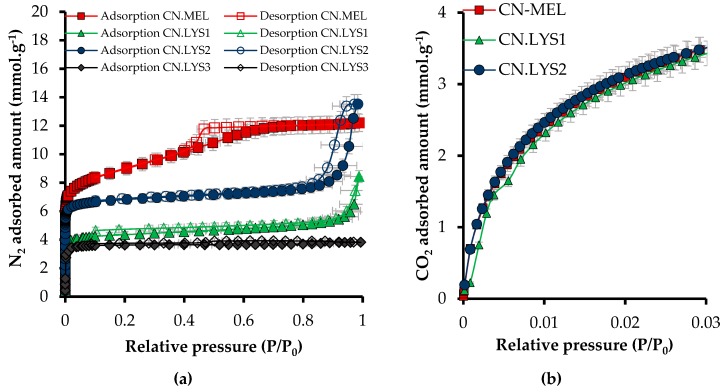
Adsorption (full symbols) and desorption (empty symbols) isotherms at atmospheric pressure for nanoparticles synthesized with melamine (CN.MEL) and L-lysine (CN.LYS). (**a**) N_2_ at −196 °C and (**b**) CO_2_ at 0 °C.

The adsorption and desorption isotherms (N_2_ at −196 °C and CO_2_ at 0 °C) for nanomaterials synthesized with melamine (CN.MEL) and L-lysine (CN.LYS) are presented in Figure 3, and the textural parameters obtained from adsorption isotherms are presented in Table 3.

The micropore and the mesopore fractions of the CN.MEL and CN.LYS2 materials were similar (67–64% and 33–36%, respectively) although their total pore volumes (at *P*/*P*_0_ = 0.95) were rather different. Both CN.LYS1 and CN.LYS3 exhibited higher micropore fractions (72.7 and 92.3%, respectively), but had lower values of *A*_BET_ and total pore volumes than those of CN.MEL and CN.LYS2 materials (Figure 3). The mesoporous volumen of CN.MEL is evidenced by the hysteresis loop in the range of relative pressures between 0.46 and 0.66. In the CN.LYS series, only CN.LYS2 was also mesoporous, but with a different structure as deduced from the different shape of the hysteresis loop, occurring at higher relative pressure. The CN.LYS1 texture was moderately mesoporous (28.6%). After the first dilution, the CN.LYS2 texture was a little more mesoporous (36.6%), but after the third dilution, CN.LYS3 had a predominantly microporous texture as evidenced by the type Ia of its N_2_ isotherm. The adsorption capacity of CO_2_ at 0 °C was as expected according to the range reported in the literature, but the synthesis process reported in this study was easier than many others reported so far [16,43,57]. The experimental development considered important parameters for a possible industrial application, such as operation at atmospheric pressure, relatively low temperature (60 °C), and relatively low time (1 h) before carbonization. The carbon nanospheres are usually synthesized by methods that demand greater energy and time [29].

**Figure 4:**
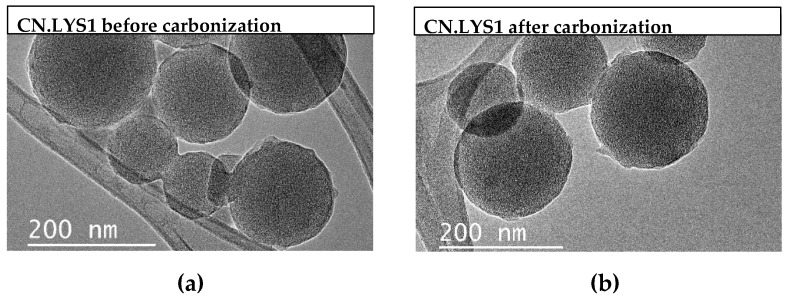

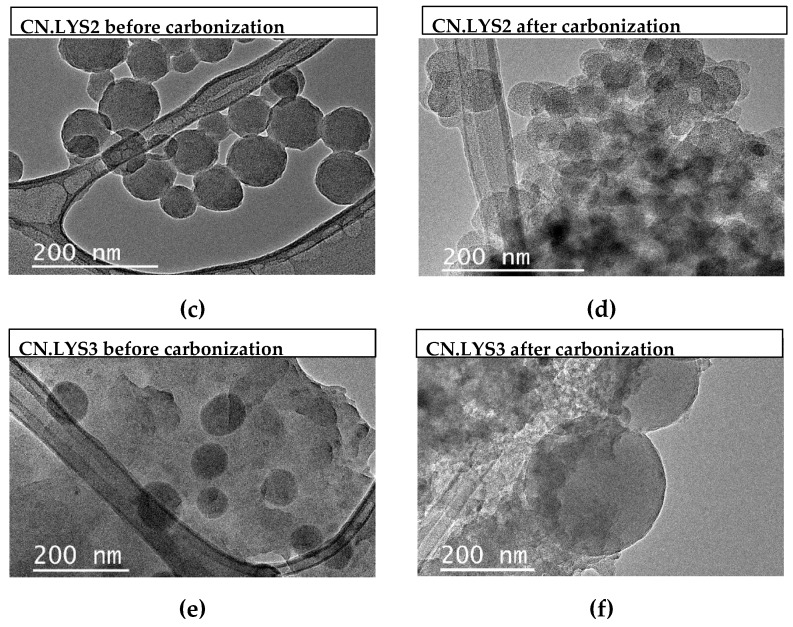
TEM images of carbon nanospheres synthesized with L-lysine. (**a**) Gel.LYS1, (**b**) CN.LYS1, (**c**) Gel.LYS2, (**d**) CN.LYS2, (**e**) Gel.LYS3, and (**f**) CN.LYS3.

In order to provide explanations of the aforementioned trends, Figure 4 presents TEM pictures of Gel.LYS (materials before carbonization) and CN.LYS (materials after carbonization).

Figure 4a,b present CN.LYS1 before and after carbonization, respectively. Here a mixture of larger and smaller nanospheres can be seen. Figure 4c,d present smaller particles (approximately 50 nm) for CN.LYS2 before and after carbonization. These particles are more transparent than those of the CN.LYS1 and CN.LYS3 materials, due to the more mesoporous texture of the CN.LYS2 material. Figure 4e,f present CN.LYS3 before and after carbonization. It can be seen that a gel was formed around the spheres. The L-lysine acts as a catalyst in the reaction, and therefore, if its amount is limited for the third dilution process, it might be the reason for the formation of a gel coating the spheres instead of producing more nanospheres, which obstructs the porous structure of CN.LYS3. This would affect the results presented in Figure 3 and Table 3 for CN.LYS3. The reproducibility for CN.LYS is significant, the variation of size and CO_2_ capacity is less than 1%.

**Figure 5:**
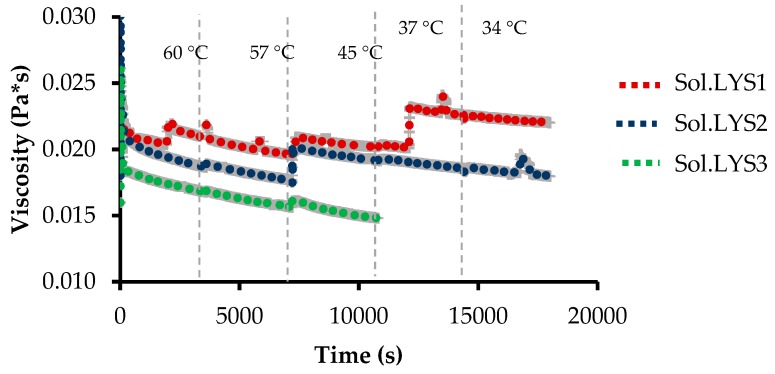
Rheological analysis of synthesis solutions with L-Lysine (Sol.LYS) in the same thermal conditions but with different resorcinol/water molar ratios: 1:2778 (Sol.LYS1), 1:5556 (Sol.LYS2), and 1:11112 (Sol.LYS3).

Figure 5 presents the rheological behavior of the synthesis solution/colloid/suspension (Sol.LYS1 without dilution, Sol.LYS2 for dilution 1, and Sol.LYS3 for dilution 2). The changes in temperature and the stirring were chosen to reproduce the conditions of synthesis (60 °C for 1 h and after cooling at room temperature). The cooling stage was controlled in the rheometer. The viscosity has changed during synthesis because the reactive system underwent polymerization and condensation-related changes to form the nanospheres, so that the system passed from the solution to the colloid and the suspension. The differences in viscosity were related to the concentration of reagents in the solutions. The third solution, Sol.LYS3, presented fewer changes during the synthesis process because the system had a lower concentration of reagents as presented in Figure 4.

Based on the particle size analysis, porous texture, nitrogen content, and adsorbed amount of CO_2_ (at 0 °C and up to 1 bar), the CN.LYS material was selected to perform the adsorption tests in conditions closer to those of the reservoir. Despite the high CO_2_ adsorption capacities exhibited by the CN.MEL material, its size and shape would not allow its application in real reservoir conditions. Besides, its synthesis process was more complex, with a higher number of steps and a higher consumption of energy and time, which were not compensated by significantly better physicochemical properties regarding the CN.LYS.

**Figure 6:**
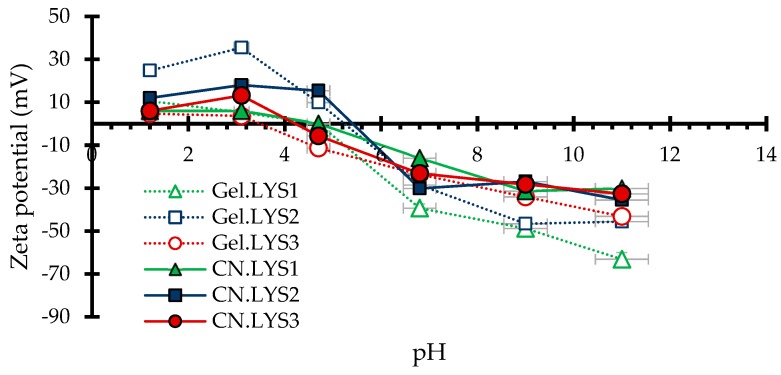
Zeta potential for carbon nanoparticles synthesized with L-lysine.

To analyze the behavior of the nanoparticles in aqueous medium, Table 4 presents the mean particle size of nanomaterials (CN.LYS) and Figure 6 presents their zeta potentials. According to the Stokes–Einstein equation, the diffusion coefficient is inversely proportional to particle size or hydrodynamic diameter; therefore, it is possible to analyze whether the nanoparticles could interact to form aggregates. Precipitation is only possible if the aggregates are big enough. Another important concept is zeta potential; if zeta potential is high (negative or positive), the particles are stable due to the high electrostatic repulsion between them. On the contrary, a low zeta potential (approaching zero) increases the probability of particles colliding, and thus forming aggregates. The hydrodynamic diameter was calculated for nanoparticles in water (at pH 5.8) and ethanol (at pH 7). Aggregate size was less in ethanol (Table 4) because the pH affects the behavior in solution (Figure 6). However, the results for nanoparticles suspended in water was close those obtained for nanoparticles suspended in ethanol. For an industrial application and injection into the porous medium, the most economical way is suspension in water.

For CN.LYS1 and CN.LYS3, a pH higher than 7 was better for rocks impregnation because the zeta potential was farther from zero. For CN.LYS2, a pH higher than 7 or lower than 4.7 was better for rocks impregnation. Gel.LYS2 presented the highest values of zeta potential at pH below 4, increasing the natural precipitation time of nanoparticles after the synthesis process.

**Figure 7:**
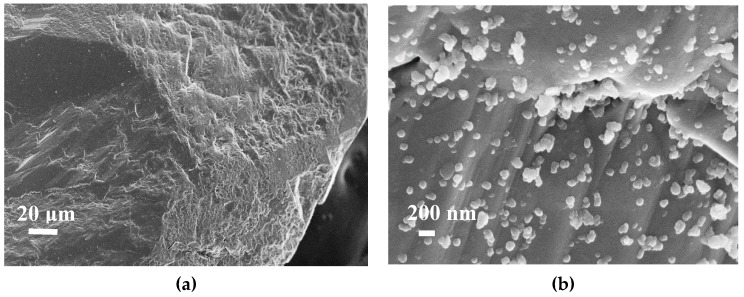
SEM images of (**a**) sandstone and (**b**) sandstone impregnated with a mass fraction of 20% of CN.LYS2.

Figure 7 presents SEM images of the sandstone surface before (Figure 7a) and after (Figure 7b,c) the impregnation step. Impregnation was achieved in water because of its lower cost and non-hazardous nature, making it ideal for industrial applications. The distribution of CN.LYS2 particles was homogeneously distributed on the surface (Figure 7b). The size of the aggregates was between 100 and 200 nm (Figure 7b). After one year, the sandstone continued to be impregnated, without showing any disintegration of nanoparticles from the sandstone surface. By thermogravimetric analysis, variations of less than 5% of the percentage of impregnation were obtained. This can also be related to the impregnation method without stirring, which might produce zones of lower nanoparticle concentration.

The sandstone presented an *A*_BET_ of 0.4 m^2^ g^−1^, and its CO_2_ adsorption capacity could not be measured using conventional methods (<0.0013 mmol g^−1^ at 0 °C and atmospheric pressure). Sandstone is mainly composed of silica, which has an acidic character as the CO_2_ molecule. Consequently, if the specific area of the sandstone is low, its CO_2_ adsorption capacity is even lower than that which might be expected for this specific area.

The sandstone was impregnated at a low nanoparticle concentration to evaluate its economic feasibility at the industrial level. The sandstone impregnated with mass fractions of 0.1 and 0.01% did not show a significant increase in its surface properties, unlike samples with higher mass fractions as shown in Table 5. The textural parameters of the impregnated sandstones were indeed improved as the percentage of nanoparticles on their surface increased. At a mass fraction of 20%, *A*_BET_ and *V*_0.95_ increased by factors as high as 225 and 670, respectively.

**Figure 8:**
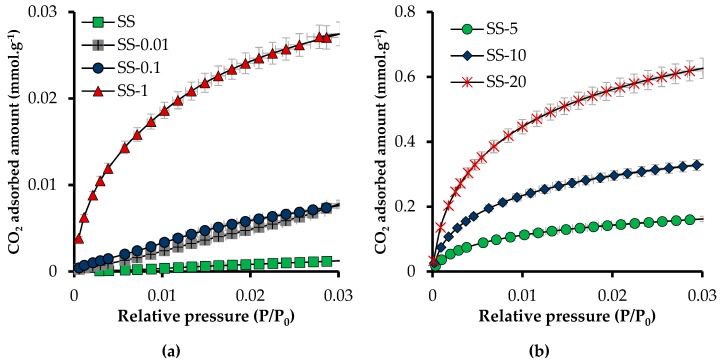
Adsorption isotherms of CO_2_ at atmospheric pressure and 0 °C. (**a**) Mass fractions ≤1% and (**b**) mass fractions ≥5%.

Figure 8 shows adsorption isotherms of CO_2_ at atmospheric pressure and 0 °C, for raw sandstone and sandstone impregnated at mass fractions of 0.01, 0.1, 1, 5, 10, and 20% of CN.LYS2. In addition, it shows the slope changes related to the affinity between the adsorbent medium and the adsorbate. The materials did not have significant affinity with CO_2_ at mass fractions of 0.01 and 0.1% and without CN.LYS2. The affinity and adsorption capacity increased with the percentage of nanoparticles in the system. The latter presented a different behavior above a mass fraction of 1%. Indeed, at a mass fraction of 1%, the adsorption capacity was increased by a factor 21 with respect to raw sandstone, although the value was still low, 0.03 mmol g^−1^. At a mass fraction of 20%, the value was far higher, 0.63 mmol g^−1^, corresponding to an increment factor of 499. Different materials have been reported in the literature [16,43] with specific surface modifications to increase the adsorption capacity of CO_2_, but the value of adsorption capacity was similar to that of sandstone by adding a mass fraction of 10 or 20% of nanoparticles.

The effect of the impregnation method on the CO_2_ adsorption capacity at atmospheric pressure and 0°C was then evaluated. As explained before, immersion and soaking were achieved using two different sets of conditions: (i) 6 h and 600 rpm and (ii) 24 h without stirring. Increasing the soaking time by 18 h improved the adsorption capacity by more than 25% (Table 6). The values presented in Figure 8, adsorption isotherms of CO_2_ at atmospheric pressure and 0 °C, thus correspond to Conditions 2.

To evaluate the possible synergistic behavior between NC.LYS2 and sandstone, the theoretical and experimental values of the CO_2_ adsorption capacity are presented in Table 7. Theoretical values were calculated by assuming a linear relationship and taking into account the CO_2_ adsorption capacities and the percentages of each solid. The difference between theoretical and experimental values ranged from 5 to 10%, which corresponds to the experimental error given the inaccuracy in the measurement of the very low CO_2_ adsorption capacity of the sandstone. The differences could also be related to the segregation of nanoparticles during the impregnation process, the nanoparticles not being homogeneously distributed on the surface of the sandstone.

### 3.2. High-Pressure Adsorption Tests

#### 3.2.1. Pure CO_2_ Adsorption at High Pressure–Manometric Measurement Method

**Figure 9:**
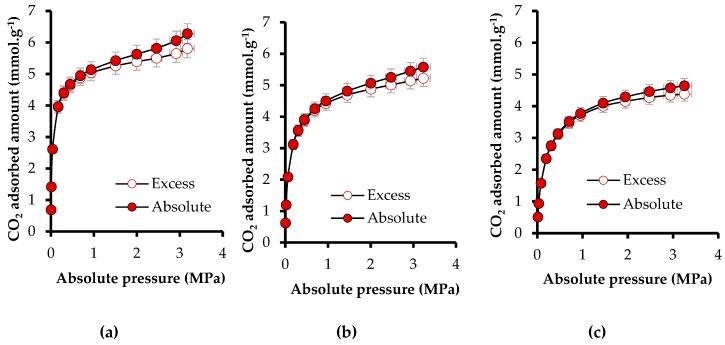

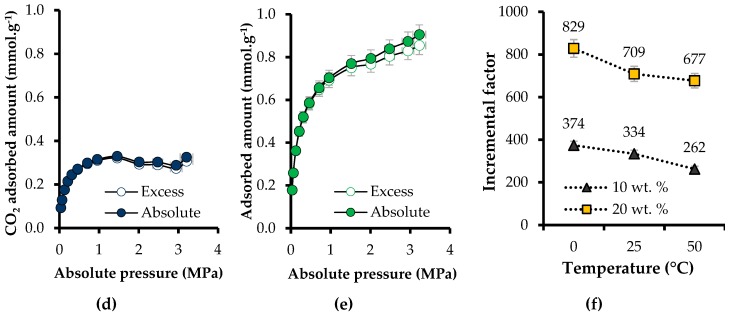
Adsorption isotherms of CO_2_ at high pressure (3 × 10^−3^ up to 3.0 MPa) of CN.LYS2 at (**a**) 0 °C, (**b**) 25 °C, and (**c**) 50 °C; (**d**) sandstone impregnated with a mass fraction of 10% at 50 °C, (**e**) sandstone impregnated with a mass fraction of 20% at 50 °C. (**f**) Relationship between the impregnation percentages (mass fractions of 10 and 20%) and the adsorption capacity of CO_2_ at 3 MPa and 0, 25, and 50 °C.

The e-CCS process requires evaluating the behavior of the materials at high pressure (up to 3 MPa) and at the temperature of a hypothetical reservoir (50 °C). Figure 9a–c present the absolute CO_2_ adsorbed amount and the excess amount for CN.LYS2 at 0, 25, and 50 °C. The difference between excess and absolute amounts appeared above 1 MPa, and represented 8.3% at 0 °C, 6.7% at 25 °C, and 5.9% at 50 °C. This difference was lower when the temperature increased. Such a trend is consistent with the fact that, at similar pressure, the density of the bulk phase decreases when the temperature increases. The high-pressure intrinsic CO_2_ adsorption capacity of sandstone without impregnation had a negligible effect on the measurement. Therefore, the evaluation was only carried out for sandstone impregnated at mass fractions of 10% and 20% (Figure 9d,e). The difference between excess and absolute adsorbed amounts of impregnated sandstone was similar to that of CN.LYS2, stranded out after 1 MPa, and represented less than 10%, on average.

To observe the pressure effect on the adsorption capacity of CN.LYS2, the *N*_ads_ at atmospheric pressure and 0 °C (3.48 mmol g^−1^) was compared to *N*_ads_ at 3 MPa and 0 °C (5.80 mmol g^−1^). The increase of pressure produced an increase of *N*_ads_ of 66.6%, indicating physisorption as the main adsorption mechanism. At 50 °C, as expected, the adsorption capacity decreased by 20% due to the exothermic character of adsorption. The obtained adsorption capacity is competitive compared to other results reported for nanomaterials under similar conditions [43,44,58]. The effect of pressure on the impregnated sandstone was also observed by comparing *N*_ads_ at atmospheric pressure and 0 °C (0.34 mmol g^−1^ for SS-10 and 0.63 mmol g^−1^ for SS-20) to *N*_ads_ at 3 MPa and 0 °C (0.47 mmol g^−1^ for SS-10 and 1.04 mmol g^−1^ for SS-20). The corresponding increases were 38.2% (SS-10) and 66.0% (SS-20), respectively. The maximum *N*_ads_ under reservoir conditions was 0.85 mmol g^−1^ at a mass fraction of 20% of CN.LYS2.

**Figure A1:**
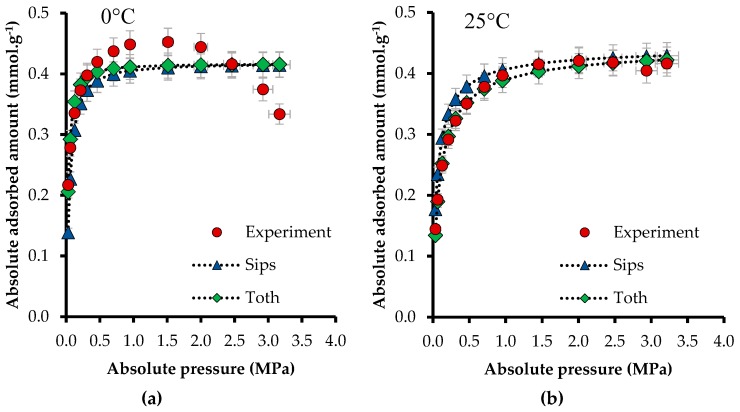

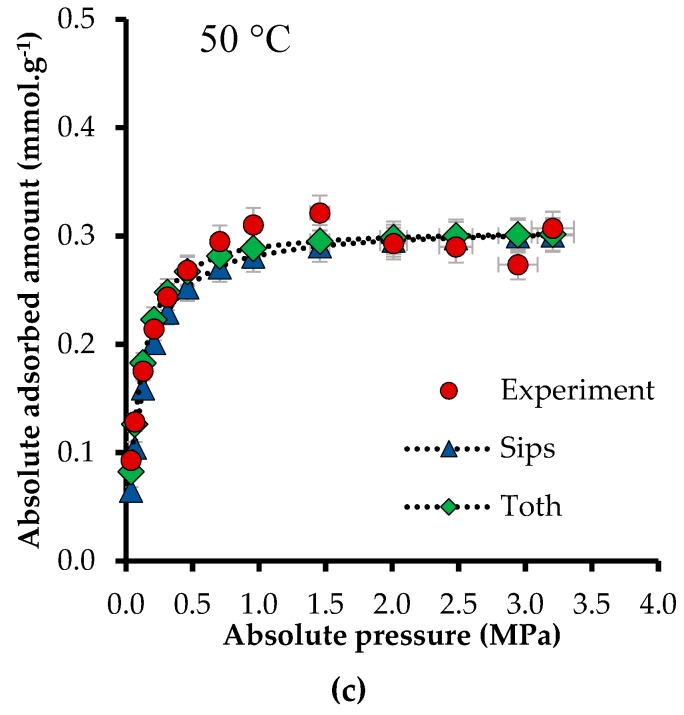
Adsorption isotherms fitted by the Sips and Toth models for sandstone impregnated with a mass fraction of 10% of carbon nanospheres synthesized with L-Lysine (CN.LYS2) at (**a**) 0° C, (**b**) 25 °C, and (**c**) 50 °C.

**Figure A2:**
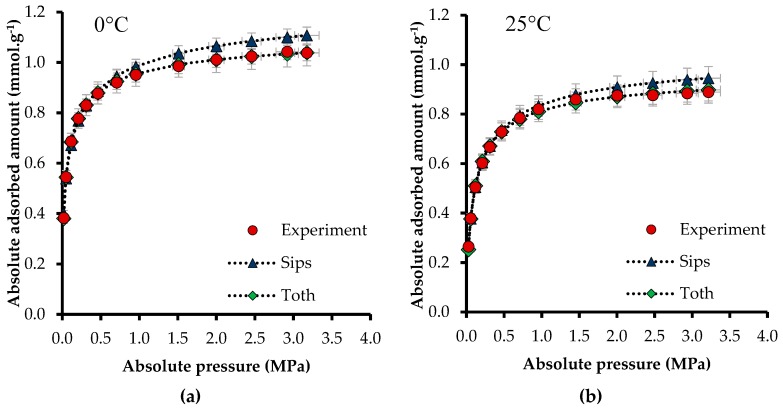

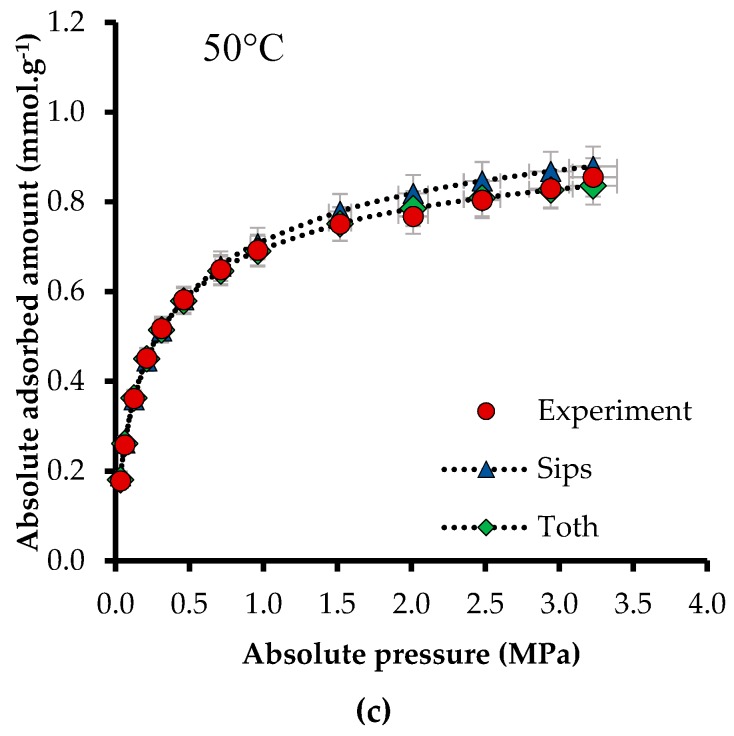
Adsorption isotherms fitted the Sips and Toth models for sandstone impregnated with a mass fraction of 20% of carbon nanospheres synthesized with L-Lysine (CN.LYS2) at (**a**) 0 °C, (**b**) 25 °C, and (**c**) 50 °C.

**Figure A3:**
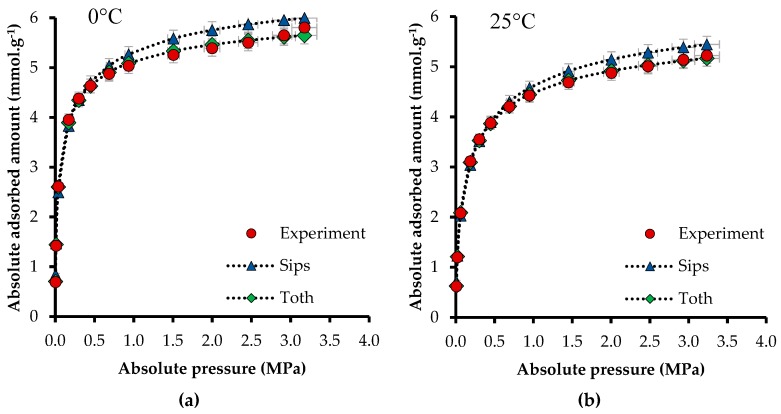

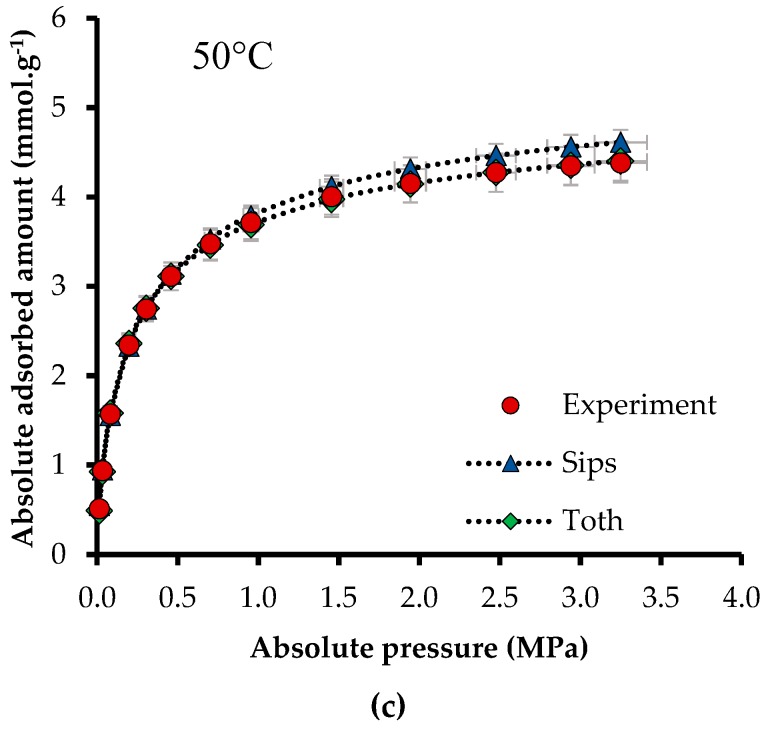
Adsorption isotherms fitted by the Sips and Toth models for carbon nanospheres synthesized with L-lysine (CN.LYS2) at (**a**) 0 °C, (**b**) 25 °C, and (**c**) 50 °C.

Figure 9e presents incremental factors comparing *N*_ads_ for sandstone without impregnation to *N*_ads_ after impregnation with mass fractions of 10 and 20% at 3 MPa and 0, 25, and 50 °C. In the conditions of a shallow reservoir (50 °C and 3.0 MPa), the incremental factor was 677 for SS-20. For the e-CCS process, the pressure could increase up 6.0 MPa so that CO_2_ is still in vapor phase, which allows a higher adsorbed amount. Appendix A presents the fit of the Sips and Toth models to the CO_2_ adsorption isotherms at high pressure and at 0, 25, and 50°C for CN.LYS2, SS-10, and SS-20 (Figure A1, Figure A2 and Figure A3, respectively). As expected, the Toth and Sips models led to very good fits: R^2^ > 0.99 for CN.LYS2, SS-10, and SS-20, and R^2^ > 0.75 for SS-10 at 0 °C. In the latter case, the concavity of the isotherm was mainly due to the very rapid increase of the bulk density as a function of pressure with respect to the increase of the density of the adsorbed phase at pressure higher than 1.5 MPa.

**Figure 10:**
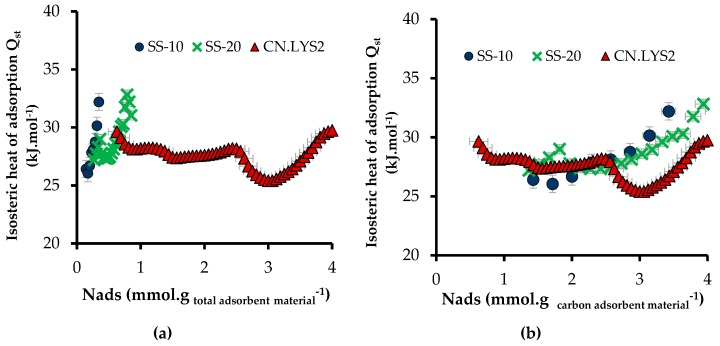
Isosteric heat of adsorption of CN.LYS2 and sandstone impregnated with mass fractions of 10 and 20%, as a function of the adsorbed CO_2_ amount expressed: (**a**) in mmol per total amount of adsorbent material (sandstone and CN.LYS2) and (**b**) in mmol per amount of carbon adsorbent material.

Figure 10a presents the isosteric heat of adsorption (*Q*_st_) of CN.LYS2 and of sandstone impregnated with mass fractions of 10 and 20%, as a function of *N*_ads_ expressed in mmol per gram of total adsorbent material. *N*_ads_ for SS-10 and SS-20 was small, and thus, Figure 10b presents *Q*_st_ as a function of *N*_ads_ in mmol per gram of carbon adsorbent material. The values of *Q*_st_ varied from 25 to 33 kJ mol^−1^, which indicates a strong interaction in the adsorption system (high affinity). The interactions with the carbon porous structure could be increased by nitrogen-containing groups that are present onto the carbon surface. Nitrogen groups can indeed promote interactions between CO_2_ and the substrate. The values of *Q*_st_ compare favorably with those reported in the literature for different materials doped or not with nitrogen (20–25 kJ mol^−1^, on average) [45,59,60,61].

The isosteric heat of adsorption of CN.LYS2 presented two distinct behaviors, whether *N*_ads_ was either (i) lower or (ii) higher than 3 mmol g_carbonadsorbentmaterial_^−1^. For case (i), *Q*_st_ decreased with *N*_ads_ due to the increasing distance between the last adsorbed layer and the carbon surface, thus decreasing the molecular interactions (Figure 10b). For case (ii), i.e., when *N*_ads_ was higher than 3 mmol g_carbonadsorbentmaterial_^−1^, *Q*_st_ increased due to the increased interactions between adsorbate molecules [61]. For SS-10 and SS-20, the value of *Q*_st_ also increased with *N*_ads_. Because the interactions between sandstone (silica) and CO_2_ were weak and because the contribution of the surface area associated to the carbon material was lower than for CN.LYS2 alone, it can be assumed again that the interactions between the adsorbed gas layers prevailed [61].

#### 3.2.2. CO_2_ and N_2_ Adsorption at High Pressure–Gravimetric Measurement Method

Using the PyIAST application and using the pure-components adsorption isotherms obtained by experimental tests, it is possible to characterize the behavior of each component and to predict the adsorbed amount of each component present in a mixture [56]. It is, thus, possible to obtain the adsorbed amount at a constant temperature by varying the concentration of the components (at constant pressure) or the pressure (at constant concentrations). Initially, it is necessary to use the “isothermal interpolator” to generate data points that follow a given isothermal model, avoiding the search for an appropriate analytical model and examining the quality of its fit to the data (i.e., Langmuir, Freundlich or Toth, among others) [56]. The isothermal interpolator is a tool included in the PyIAST package. After that, PyIAST takes the interpolated data for its calculations. Calculations are done using a predesigned routine [56].

In the present case, the experimental data of each pure component (CO_2_ and N_2_) were obtained by HP-TGA at 50 °C, between 0.1 and 2.5 MPa, and with a flow of CO_2_ or N_2_ (50 mL min^−1^ up to 1.0 MPa and 70 mL min^−1^ up to 2.5 MPa). The prediction was calculated at 50 °C by varying: (1) the system pressure from 0.1 to 2.5 MPa at constant CO_2_ concentration of 20% and (2) the concentration of CO_2_, from 5 to 100% at a constant pressure of 2.5 MPa. The evaluated materials were CN.LYS2 and sandstone from a real reservoir (RS) impregnated with a mass fraction of 20% of CN.LYS2. It is important to mention that it might be possible to obtain considerable adsorbed quantities for a cleaning and adsorption balance of more than 12–24 h, because the nanomaterials (main adsorbent) are micro/mesoporous. A longer cleaning time thus allows eliminating adsorbed gases and moisture. In the same way, an adequate equilibrium time allows for greater diffusion of the gas into the porous structure and greater interactions with the material, which would allow a higher adsorbed amount. Therefore, to analyze the selectivity, a shorter time was used (cleaning and adsorption equilibrium time of 2 h at each pressure).

**Figure 11:**
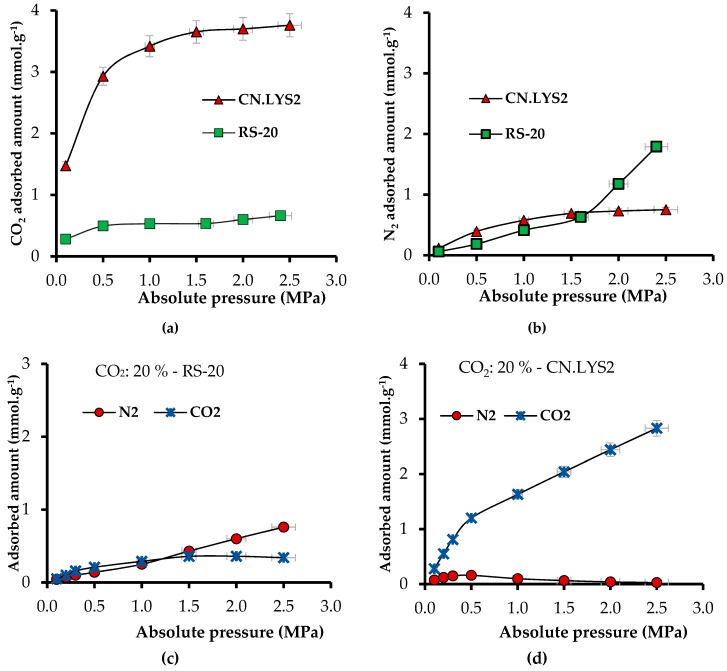

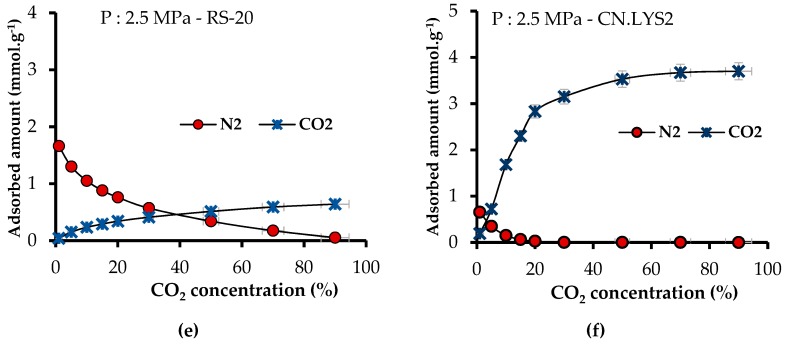
Adsorption isotherm of CN.LYS2 and RS-20 at 50 °C between 0.1 and 2.5 MPa for (**a**) pure CO_2_ and (**b**) pure N_2_. Adsorption isotherm simulating a N_2_/CO_2_ mixture at 50 °C, constant CO_2_ concentration of 20% and varying the pressure for (**c**) RS-20 and (**d**) CN.LYS2; and varying the CO_2_ concentration at constant pressure of 2.5 MPa for (**e**) RS-20 and (**f**) CN.LYS2.

It was not possible to obtain the CO_2_ isotherm for RS because the latter had a too low surface area, so the maximum value for RS at 50 °C and 2.5 MPa was 0.033 mmol g^−1^. For RS, the adsorbed amount of N_2_ was 3.19 mmol g^−1^ at 50 °C and 2.5 MPa. Figure 11a,b present the CO_2_ and N_2_ adsorption isotherms under continuous gas flow for CN.LYS2 and RS-20. The increment factor of *N*_ads_ for RS-20 with respect to RS was 19 (0.66 mmol g^−1^ at 50 °C and 2.5 MPa). In addition, the theoretical value of *N*_ads_ for RS with a mass fraction of 20% of CN.LYS was 0.78 mmol g^−1^ but the experimental value was 0.66 mmol g^−1^ (i.e., 85% of the theoretical one).

Figure 11c,d present the simulated adsorbed amount for a N_2_/CO_2_ mixture at constant CO_2_ concentration of 20% and at 50 °C by varying the pressure for RS-20 and CN.LYS2, respectively. For RS-20, the affinity for CO_2_ was higher (P < 1 MPa) compared to RS without impregnation, but the *N*_ads_ for N_2_ was superior (123.5%) to that for CO_2_ (Figure 11c). For RS-20, the CO_2_ isotherm obtained a form that would be impossible to obtain without impregnation due to the lack of surface area and molecular interactions. The isotherm for CN.LYS2 showed a higher affinity for CO_2_ and a correspondingly higher *N*_ads_ than for N_2_, which, above 0.5 MPa, decreased considerably (Figure 11d). Similar conditions occurred when the CO_2_ concentration was varied at 2.5 MPa and 50 °C (Figure 11e,f), CN.LYS2 had more affinity for CO_2_ than RS-20, and CN.LYS2 had a considerably higher *N*_ads_ for CO_2_ than for N_2_. Figure 11e shows the increment of *N*_ads_ only for CO_2_ while the CO_2_ concentration was increased in RS-20; at low concentrations of CO_2_ (< 30%), the *N*_ads_ for N_2_ was higher. For CN.LYS2 at low concentrations of CO_2_ (1%), the slope increment was significant, indicating a higher affinity for CO_2_ at low concentrations.

From Figure 11, it can be concluded that the pyIAST is a useful tool because it is possible to simulate the behavior of adsorption systems from some pure gas adsorption data.

## 4. Conclusions

To the best of our knowledge, this is the first study using nanoparticles to modify the CO_2_ adsorption capacities of a reservoir in a carbon capture and storage (CCS) process. Moreover, this is the first research proposing a possible new configuration of the CCS process in which the storage is performed in shallow reservoirs (less than 300 m). We called it enhanced CCS (e-CCS), for which the main advantage is that the CO_2_ capture/separation step is removed, and the flue gas is injected directly into shallow deposits, where the CO_2_ is gaseous and where the adsorption phenomena control capture and storage.

Nitrogen-rich carbon nanospheres allowed increasing the adsorption capacity by 67,700% with a mass fraction of only 20% under realistic reservoir conditions (50 °C and 3 MPa). This was possible thanks to the higher surface area and to the favorable chemical composition, which promoted the capture and storage of CO_2_. These N-doped carbon nanospheres, synthesized by a simple process, had competitive CO_2_ capture performances compared to other special materials reported in the literature. Therefore, this research opens an interesting line of research that would expand knowledge in the field of carbon nanospheres for application in the adsorption and geological storage of CO_2_.

## Figures and Tables

**Table 1:** Models for adsorption isotherms.

Model	Equations	Parameters
Sips	(7)	*N*_ads_ (mmol g^−1^) is the adsorbed amount, *N*_m_ (mmol g^−1^) is the adsorption capacity at equilibrium, *P* (kPa) is the equilibrium pressure, and *K*_S_ and *n* are the Sips adsorption equilibrium constants, related to the affinity and the heterogeneity of the system, respectively.
Toth	(8)	*N*_ads_, *N*_m_, and *P* have the same meaning as above, and *K*_T_ and *t* are the Toth adsorption equilibrium constants, related to the affinity and the heterogeneity of the system, respectively.

**Table 2:** Ultimate analysis of nanoparticles synthesized with melamine (CN.MEL) and L-lysine (CN.LYS).

	C (Mass Fraction %)	H (Mass Fraction %)	N (Mass Fraction %)	O (Mass Fraction %)
Gel.MEL1	22.8	4.7	49.1	7.3
Gel.MEL2	55.9	5.0	9.2	31.0
CN.MEL	85.6	2.3	2.2	10.2
Gel.LYS1	59.5	6.7	5.1	31.9
Gel.LYS2	61.7	6.7	5.0	31.6
Gel.LYS3	62.7	6.4	5.0	28.3
CN.LYS1	88.6	1.7	1.7	9.9
CN.LYS2	91.1	1.7	1.9	12.0
CN.LYS3	91.9	2.1	2.2	12.8

**Table 3:** Parameters obtained from adsorption isotherms (N_2_ at −196 °C and CO_2_ at 0 °C) for nanomaterials synthesized with melamine (CN.MEL) and L-lysine (CN.LYS).

	A_BET_ (m^2^ g^−1^)	V_0.95_ (cm^3^ g^−1^)	V_mic-N2_ (cm^3^ g^−1^)	V_mic-CO2_ (cm^3^ g^−1^)	V_mes_ (cm^3^ g^−1^)	L_0_ (nm)	E_ads.CO2_ (kJ mol^−1^)
CN.MEL	713	0.42	0.28 (66.6%)	0.26	0.14	0.77	30.9
CN.LYS1	385	0.22	0.16 (72.7%)	0.18	0.06	0.84	32.4
CN.LYS2	612	0.36	0.23 (63.9%)	0.25	0.13	0.56	31.6
CN.LYS3	320	0.13	0.12 (92.3%)	-	0.01	0.62	-

**Table 4:** Mean particle size of nanomaterials in suspension, synthesized with L-lysine.

Material	*d*_p_ 50 (nm) in Water (pH 5.8)	*d*_p_ 50 (nm) in Ethanol (pH 7)
Gel.LYS1	579.4	361.5
Gel.LYS2	314.2	274.2
Gel.LYS3	1083.4	1957.1
CN.LYS1	801.1	785.9
CN.LYS2	242.6	239.9
CN.LYS3	2828.7	2587.4

**Table 5:** Parameters obtained from adsorption isotherms (N_2_ at −196 °C and CO_2_ at 0 °C) for sandstone impregnated with mass fractions of 1, 5, 10, and 20 % of CN.LYS2.

	A_BET_ (m^2^ g^−1^)	V_0.95_ (cm^3^ g^−1^)	V_mic-N2_ (cm^3^ g^−1^)	V_mic-CO2_ (cm^3^ g^−1^)	V_mes_ (cm^3^ g^−1^)	L_0_ (nm)
**SS-1**	2	0.003	0.002	0.002	0.001	0.56
**SS-5**	20	0.016	0.01	0.012	0.006	0.53
**SS-10**	49	0.035	0.021	0.023	0.014	0.51
**SS-20**	99	0.067	0.042	0.044	0.025	0.52

**Table 6:** Relationship between soaking time and adsorption capacity of CO_2_ at atmospheric pressure, 0 °C and mass fractions of 5, 10, and 20%. Conditions 1: 6 h and 600 rpm; Conditions 2: 24 h without stirring.

	5%	10%	20%
Conditions 1 (mmol g^−1^)	0.12	0.27	0.49
Conditions 2 (mmol g^−1^)	0.16	0.33	0.63
Increment (%) from conditions 1 to 2	40.5	25.4	27.2

**Table 7:** CO_2_ adsorption capacity at atmospheric pressure, 0°C, and mass fractions of 1, 5, 10, and 20 %. Theoretical and experimental values.

	1%	5%	10%	20%
Theoretical N_ads_ (mmol g^−1^)	0.036	0.175	0.349	0.697
Experimental N_ads_ (mmol g^−1^)	0.027	0.162	0.333	0.627
Relative difference (%)	23.8	7.4	4.7	10.1

## Appendix A

This section presents the adsorption isotherms fitted by the Sips and Toth models, for CO_2_ at high pressure and 0, 25, and 50 °C. This information is related to Section 3.2.1. Pure CO_2_ adsorption at high pressure–Manometric measurement method.

## References

[1] Root T.L., Price J.T., Hall K.R., Schneider S.H., Rosenzweig C., Pounds J.A. (2003). Fingerprints of global warming on wild animals and plants. Nature.

[2] Vitousek P.M. (1994). Beyond global warming: Ecology and global change. Ecology.

[3] McGlade C., Ekins P. (2015). The geographical distribution of fossil fuels unused when limiting global warming to 2 C. Nature.

[4] Harvey L.D. (2018). Global Warming.

[5] Baer H., Singer M. (2016). Global Warming and the Political Ecology of Health: Emerging Crises and Systemic Solutions.

[6] Lashof D.A., Ahuja D.R. (1990). Relative contributions of greenhouse gas emissions to global warming. Nature.

[7] Anderson T.R., Hawkins E., Jones P.D. (2016). CO_2_, the greenhouse effect and global warming: From the pioneering work of Arrhenius and Callendar to today’s Earth System Models. Endeavour.

[8] US-EPA E.P.A. (2019). Global Greenhouse Gas Emissions Data. https://www.epa.gov/ghgemissions/global-greenhouse-gas-emissions-data.

[9] Edenhofer O., Pichs-Madruga R., Sokona Y., Minx J.C., Farahani E., Kadner S., Seyboth K., Adler A., Baum I., Brunner S. (2014). Climate Change 2014, Mitigation of Climate Change. Contribution of Working Group III to the Fifth Assessment Report of the Intergovernmental Panel on Climate Change.

[10] Tan Y., Nookuea W., Li H., Thorin E., Yan J. (2016). Property impacts on Carbon Capture and Storage (CCS) processes: A review. Energy Convers. Manag..

[11] Change N.G.C. (2018). Vital Signs of the Planet.

[12] NASA (2019). Global Climate Change. Vital Signs of the Planet. https://climate.nasa.gov/vital-signs/carbon-dioxide/.

[13] Norby R.J., Luo Y. (2004). Evaluating ecosystem responses to rising atmospheric CO_2_ and global warming in a multi-factor world. New Phytol..

[14] Halmann M.M. (2018). Chemical Fixation of Carbon DioxideMethods for Recycling CO_2_ into Useful Products.

[15] Cox P.M., Betts R.A., Jones C.D., Spall S.A., Totterdell I.J. (2000). erratum: Acceleration of global warming due to carbon-cycle feedbacks in a coupled climate model. Nature.

[16] Wang J., Huang L., Yang R., Zhang Z., Wu J., Gao Y., Wang Q., O’Hare D., Zhong Z. (2014). Recent advances in solid sorbents for CO_2_ capture and new development trends. Energy Environ. Sci..

[17] Conti J., Holtberg P., Diefenderfer J., LaRose A., Turnure J.T., Westfall L. (2016). International Energy Outlook 2016 with Projections to 2040.

[18] Metz B., Davidson O., de Coninck H. (2005). Carbon Dioxide Capture and Storage: Special Report of the Intergovernmental Panel on Climate Change.

[19] Kang S.-P., Lee H. (2000). Recovery of CO_2_ from flue gas using gas hydrate: Thermodynamic verification through phase equilibrium measurements. Environ. Sci. Technol..

[20] Yang Q., Xue C., Zhong C., Chen J.F. (2007). Molecular simulation of separation of CO_2_ from flue gases in CU-BTC metal-organic framework. AIChE J..

[21] Song C., Pan W., Srimat S.T., Zheng J., Li Y., Wang Y.H., Xu B.O., Zhu Q.M. (2004). Tri-reforming of methane over Ni catalysts for CO_2_ conversion to Syngas with desired H_2_ CO ratios using flue gas of power plants without CO_2_ separation. Stud. Surf. Sci. Catal..

[22] Bui M., Adjiman C.S., Bardow A., Anthony E.J., Boston A., Brown S., Fennell P.S., Fuss S., Galindo A., Hackett L.A. (2018). Carbon capture and storage (CCS): The way forward. Energy Environ. Sci..

[23] Knorr W. (2009). Is the airborne fraction of anthropogenic CO_2_ emissions increasing?. Geophys. Res. Lett..

[24] Cook P., Causebrook R., Gale J., Michel K., Watson M. (2014). What have we learned from small-scale injection projects?. Energy Procedia.

[25] IEA I. (2011). World Energy Outlook 2011.

[26] Balat H., Öz C. (2007). Technical and Economic Aspects of Carbon Capture an Storage—A Review. Energy Explor. Exploit..

[27] Gough C. (2008). State of the art in carbon dioxide capture and storage in the UK: An experts’ review. Int. J. Greenh. Gas Control.

[28] Gough C. (2016). Carbon Capture and Its Storage: An Integrated Assessment.

[29] Bailon-García E.P.C., Agustín F., Elizabeth R.A., Francisco C.M., Franco C.A.a.C.C., Farid B. (2018). Nanoparticle Fabrication Methods. Formation Damage in Oil and Gas Reservoirs. Nanotechnology Applications for Its Inhibition/Remediation.

[30] Franco C.A.C.C., Farid B. (2018). Formation Damage in Oil and Gas Reservoirs. Nanotechnology Applications for Its Inhibition/Remediation.

[31] Franco C.A., Zabala R., Cortés F.B. (2017). Nanotechnology applied to the enhancement of oil and gas productivity and recovery of Colombian fields. J. Pet. Sci. Eng..

[32] Franco C.A., Nassar N.N., Ruiz M.A., Pereira-Almao P., Cortés F.B. (2013). Nanoparticles for inhibition of asphaltenes damage: Adsorption study and displacement test on porous media. Energy Fuels.

[33] Moncayo-Riascos I., Franco C.A., Cortés F.B. (2019). Dynamic Molecular Modeling and Experimental Approach of Fluorocarbon Surfactant-Functionalized SiO_2_ Nanoparticles for Gas-Wettability Alteration on Sandstones. J. Chem. Eng. Data.

[34] Hurtado Y., Beltrán C., Zabala R.D., Lopera S.H., Franco C.A., Nassar N.N., Cortés F.B. (2018). Effects of Surface Acidity and Polarity of SiO_2_ Nanoparticles on the Foam Stabilization Applied to Natural Gas Flooding in Tight Gas-Condensate Reservoirs. Energy Fuels.

[35] Cardona L., Arias-Madrid D., Cortés F., Lopera S., Franco C. (2018). Heavy oil upgrading and enhanced recovery in a steam injection process assisted by NiO-and PdO-Functionalized SiO_2_ nanoparticulated catalysts. Catalysts.

[36] Yang D., Wang S., Zhang Y. (2014). Analysis of CO_2_ migration during nanofluid-based supercritical CO_2_ geological storage in saline aquifers. Aerosol Air Qual. Res..

[37] Silvestre-Albero J., Reinoso F.R. (2012). Nuevos materiales de carbón para la captura de CO_2_. Boletín del Grupo Español del Carbón.

[38] Zhang X.Q., Li W.C., Lu A.H. (2015). Designed porous carbon materials for efficient CO_2_ adsorption and separation. New Carbon Mater..

[39] Bandosz T.J., Seredych M., Rodríguez-Castellón E., Cheng Y., Daemen L.L., Ramírez-Cuesta A.J. (2016). Evidence for CO_2_ reactive adsorption on nanoporous S-and N-doped carbon at ambient conditions. Carbon.

[40] Lithoxoos G.P., Labropoulos A., Peristeras L.D., Kanellopoulos N., Samios J., Economou I.G. (2010). Adsorption of N_2_, CH_4_, CO and CO_2_ gases in single walled carbon nanotubes: A combined experimental and Monte Carlo molecular simulation study. J. Supercrit. Fluids.

[41] Bikshapathi M., Sharma A., Sharma A., Verma N. (2011). Preparation of carbon molecular sieves from carbon micro and nanofibers for sequestration of CO_2_. Chem. Eng. Res. Des..

[42] Chowdhury S., Balasubramanian R. (2016). Highly efficient, rapid and selective CO_2_ capture by thermally treated graphene nanosheets. J. CO2 Util..

[43] Alonso A., Moral-Vico J., Markeb A.A., Busquets-Fité M., Komilis D., Puntes V., Sánchez A., Font X. (2017). Critical review of existing nanomaterial adsorbents to capture carbon dioxide and methane. Sci. Total Environ..

[44] Ma Y., Wang Z., Xu X., Wang J. (2017). Review on porous nanomaterials for adsorption and photocatalytic conversion of CO_2_. Chin. J. Catal..

[45] Babu D.J., Bruns M., Schneider R., Gerthsen D., Schneider J.J. (2017). Understanding the influence of N-doping on the CO_2_ adsorption characteristics in carbon nanomaterials. J. Phys. Chem. C.

[46] Chen A., Li S., Yu Y., Liu L., Li Y., Wang Y., Xia K. (2016). Self-catalyzed strategy to form hollow carbon nanospheres for CO_2_ capture. Mater. Lett..

[47] Heydari-Gorji A., Belmabkhout Y., Sayari A. (2011). Degradation of amine-supported CO_2_ adsorbents in the presence of oxygen-containing gases. Microporous Mesoporous Mater..

[48] Dong Y.-R., Nishiyama N., Egashira Y., Ueyama K. (2008). Basic Amid Acid-Assisted Synthesis of Resorcinol−Formaldehyde Polymer and Carbon Nanospheres. Ind. Eng. Chem. Res..

[49] Bai X., Li J., Cao C., Hussain S. (2011). Solvothermal synthesis of the special shape (deformable) hollow g-C_3_N_4_ nanospheres. Mater. Lett..

[50] Franco-Aguirre M., Zabala R.D., Lopera S.H., Franco C.A., Cortés F.B. (2018). Interaction of anionic surfactant-nanoparticles for gas-Wettability alteration of sandstone in tight gas-condensate reservoirs. J. Nat. Gas Sci. Eng..

[51] Schaefer S., Fierro V., Izquierdo M.T., Celzard A. (2016). Assessment of hydrogen storage in activated carbons produced from hydrothermally treated organic materials. Int. J. Hydrog. Energy.

[52] Schaefer S., Fierro V., Szczurek A., Izquierdo M.T., Celzard A. (2016). Physisorption, chemisorption and spill-over contributions to hydrogen storage. Int. J. Hydrog. Energy.

[53] Tzabar N., Brake H.T. (2016). Adsorption isotherms and Sips models of nitrogen, methane, ethane, and propane on commercial activated carbons and polyvinylidene chloride. Adsorption.

[54] Álvarez-Gutiérrez N., Gil M.V., Rubiera F., Pevida C. (2016). Adsorption performance indicators for the CO_2_/CH_4_ separation: Application to biomass-based activated carbons. Fuel Process. Technol..

[55] Abdeljaoued A., Querejeta N., Durán I., Álvarez-Gutiérrez N., Pevida C., Chahbani M. (2018). Preparation and Evaluation of a Coconut Shell-Based Activated Carbon for CO_2_/CH_4_ Separation. Energies.

[56] Simon C.M., Smit B., Haranczyk M. (2016). pyIAST: Ideal adsorbed solution theory (IAST) Python package. Comput. Phys. Commun..

[57] Cavenati S., Grande C.A., Rodrigues A.E. (2004). Adsorption equilibrium of methane, carbon dioxide, and nitrogen on zeolite 13X at high pressures. J. Chem. Eng. Data.

[58] Himeno S., Tomita T., Suzuki K., Yoshida S. (2007). Characterization and selectivity for methane and carbon dioxide adsorption on the all-silica DD3R zeolite. Microporous Mesoporous Mater..

[59] Dunne J., Mariwala R., Rao M., Sircar S., Gorte R.J., Myers A.L. (1996). Calorimetric heats of adsorption and adsorption isotherms. 1. O_2_, N_2_, Ar, CO_2_, CH_4_, C_2_H_6_, and SF6 on silicalite. Langmuir.

[60] Siriwardane R.V., Shen M.S., Fisher E.P., Poston J.A. (2001). Adsorption of CO_2_ on molecular sieves and activated carbon. Energy Fuels.

[61] Himeno S., Komatsu T., Fujita S. (2005). High-pressure adsorption equilibria of methane and carbon dioxide on several activated carbons. J. Chem. Eng. Data.

